# Post-Translational Modifications in Histones and Their Role in Abiotic Stress Tolerance in Plants

**DOI:** 10.3390/proteomes11040038

**Published:** 2023-11-22

**Authors:** Madhvi Sharma, Amanpreet K. Sidhu, Mahesh Kumar Samota, Mamta Gupta, Pushpendra Koli, Mukesh Choudhary

**Affiliations:** 1 Post Graduate Department of Biotechnology, Khalsa College, Amritsar 143009, India; madhvisharma413@gmail.com (M.S.); aman.preet1807@gmail.com (A.K.S.); 2 ICAR-Central Institute of Post-Harvest Engineering and Technology, Regional Station, Abohar 152116, India; 3 ICAR-Indian Institute of Maize Research, Ludhiana 141001, India; mamta14biotech@gmail.com; 4 Plant Animal Relationship Division, ICAR-Indian Grassland and Fodder Research Institute, Jhansi 284003, India; pushpendra.koli@murdoch.edu.au; 5 Post-Harvest Biosecurity, Murdoch University, Perth, WA 6150, Australia; 6 School of Agriculture and Environment, The UWA Institute of Agriculture, The University of Western Australia, Perth, WA 6009, Australia

**Keywords:** histone, ubiquitination, ChIP, methylation, stress tolerance, post-translational modifications

## Abstract

Abiotic stresses profoundly alter plant growth and development, resulting in yield losses. Plants have evolved adaptive mechanisms to combat these challenges, triggering intricate molecular responses to maintain tissue hydration and temperature stability during stress. A pivotal player in this defense is histone modification, governing gene expression in response to diverse environmental cues. Post-translational modifications (PTMs) of histone tails, including acetylation, phosphorylation, methylation, ubiquitination, and sumoylation, regulate transcription, DNA processes, and stress-related traits. This review comprehensively explores the world of PTMs of histones in plants and their vital role in imparting various abiotic stress tolerance in plants. Techniques, like chromatin immune precipitation (ChIP), ChIP-qPCR, mass spectrometry, and Cleavage Under Targets and Tag mentation, have unveiled the dynamic histone modification landscape within plant cells. The significance of PTMs in enhancing the plants’ ability to cope with abiotic stresses has also been discussed. Recent advances in PTM research shed light on the molecular basis of stress tolerance in plants. Understanding the intricate proteome complexity due to various proteoforms/protein variants is a challenging task, but emerging single-cell resolution techniques may help to address such challenges. The review provides the future prospects aimed at harnessing the full potential of PTMs for improved plant responses under changing climate change.

## 1. Introduction

Plants face formidable challenges when confronted with abiotic stresses, necessitating precise adjustments across various physiological pathways. These adaptive responses encompass critical functions, such as photosynthesis, antioxidant regulation, water uptake, ion homeostasis, and osmolyte synthesis [1,2,3,4,5,6]. In the face of environmental constraints, these morphophysiological adaptations are underpinned by an intricate network of post-translational modifications (PTMs), orchestrated by multifaceted molecular mechanisms [7].

Plant adaptation to abiotic stresses hinges on the dynamic regulation of gene expression, encompassing a multitude of genes controlled by an array of transcription factors (TFs) and chromatin-associated factors. While substantial attention has been devoted to elucidating the roles of TFs, enzymes catalyzing covalent histone modifications, and chromatin remodeling complexes, the contribution of histone chaperones remains less explored, and their significance in this context remains enigmatic. Notably, protein phosphorylation serves as a well-established mechanism for transmitting stress signals, whereas emerging modifications, like S-nitrosylation, are still in their infancy. In the realm of PTMs, ubiquitin and SUMO conjugations emerged as central regulatory processes in eukaryotes [8,9]. These modifications, however, exert distinct effects contingent upon the transcriptional or translational stage at which the targeted transcript or protein is situated. Consequently, the interplay of these diverse PTMs collectively dictates the ultimate impact on the associated cellular processes and phenotypic outcomes.

Chromatin regulation emerges as a pivotal player in governing gene expression, with DNA methylation, histone modifications, and other genome activities intricately intertwined with adaptive responses to environmental challenges in plants [10,11,12]. An array of epigenetic mechanisms, including DNA methylation, histone modifications, ATP-dependent chromatin remodeling, incorporation of histone variants, and regulation by noncoding RNA, orchestrate the structure and function of chromatin [13,14]. Specifically, methylation, acetylation, phosphorylation, ubiquitination, and sumoylation represent a subset of PTMs occurring on the N-terminal tails of histone proteins. These modifications, collectively referred to as the “histone code,” are pivotal in establishing and perpetuating epigenetic memory, profoundly influencing chromatin structure and gene expression [15,16].

Within the intricate world of chromatin, the configuration significantly influences genome expression, largely regulated by the interplay of DNA methylation machinery and histone chaperones, also known as nucleosome assembly/disassembly factors. They facilitate nucleosome assembly and disassembly, impacting replication-dependent and replication-independent processes and collaborate with free histones to prevent indiscriminate histone–DNA interactions. Hence, histone chaperones play a critical role in modulating histone availability and the incorporation into nucleosomes [17,18].

**Figure 1:**
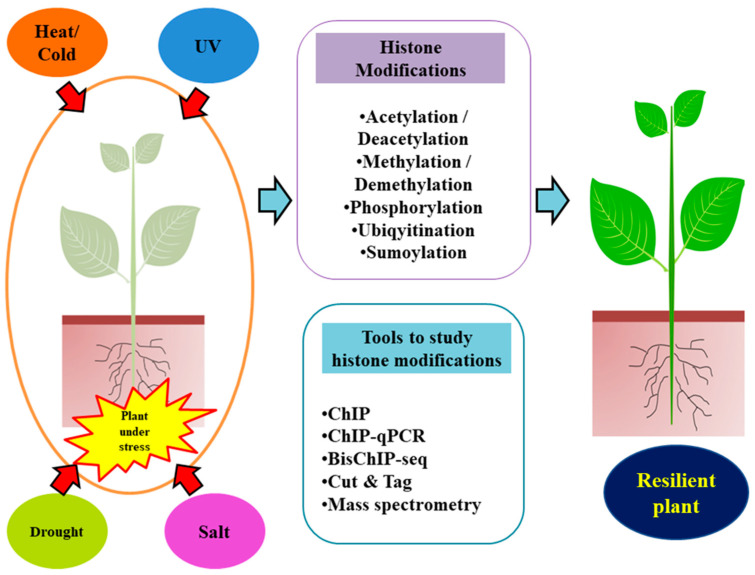
Plants are exposed to many abiotic stresses due to unpredictable climate changes, including cold and hot temperatures, drought, salinity, and ultraviolet radiation, all of which affect their productivity. Dynamic changes in histone modifications are important for the regulation of genes during environmental stress.

In summary, this multifaceted interplay of plant responses to abiotic stresses, encompassing diverse pathways, PTMs, and chromatin dynamics, underscores the intricate web of adaptations crucial for plant survival in challenging environments (Figure 1). This review will delve deeper into the roles and significance of histone chaperones within this complex landscape.

## 2. Epigenetic Memory and Chromatin Dynamics in Plant Stress Responses

**Figure 2:**
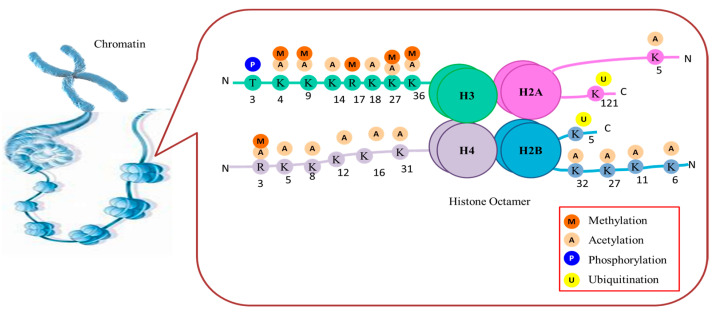
Histone modifications at different polypeptide amino acid residue locations and the group added by the different enzymes.

Plants often experience unfavorable environmental conditions in growing habitats. Depending on the stress response, plants can retain information for a time after a previous stress (known as stress memory), so they can adapt more quickly to the same adversity in the future [19]. Epigenetic regulation is closely associated with the development of stress memories [19,20,21]. Research showed that stress treatment can alter the chromatin status of genes that respond to stress, and these changes persist after recovery and even in progeny [22,23,24]. Several factors play a role in regulating gene expression in eukaryotic cells, including the dynamic environment of chromatin. Epigenetic mechanisms, including covalent modifications to DNA and histone tails, are crucial for inducing favorable chromatin states that enable gene expression in response to stress and hence bestowing the plants with better adaptation. Several epigenetic factors cause chromatin modifications on exposure to various abiotic stresses in plants [25]. Each nucleosome contains a basic core histone octamer composed of four types of histone proteins, namely H2A, H2B, H3, and H4. In addition to these core histones, many histone variants are also reported. To date, only one variant is observed for H4, whereas several variants are encountered for H2A, H2B, and H3. These variants are believed to enhance the dynamics of nucleosome, diversity and play a crucial role in epigenetic genome regulation (Figure 2). The study of these variants can provide various clues in understanding the mechanism behind epigenetic genome regulation.

The majority of histone modifications take place at the N-terminal coil known as the histone tail rather than the globular C-terminal domain. Many basic amino acid residues, such as arginine and lysine, can be found in high concentrations in histones. The amino acid residues in histone tails can change chemically through acetylation, methylation, phosphorylation, and ubiquitination. The aforementioned modifications are believed to influence the functioning of genes located in proximity to core histones. Most histone-modifying enzymes are remarkably conserved across the plant realm, including well-researched and defined histone modifiers, such as histone methyltransferases (HMTs), histone demethylases (HDMs), histone deacetylases (HDACs), and histone acetyltransferases (HATs). Other less-studied enzymes include kinases, arginine deiminases, lysine- and arginine-specific methyltransferases, ubiquitinates, lysine and arginine-specific demethylases, and deubiquitinases. While there are studies on histone modifications and stress response in plants, this research seems to delve into the specific mechanisms and examples of how histone modifications influence stress tolerance in different plant species.

## 3. Molecular Sculptors: Types of Histone Post-Translational Modifications

### 3.1. Acetylation and Deacetylation

Histone acetylation involves a covalent alteration that enables the transfer of acetyl groups from acetyl CoA to the ε-amino group of lysine residues within histone molecules. This modification leads to the neutralization of lysine’s positive charge, subsequently diminishing the binding affinity between the modified histone and DNA [26,27]. Conversely, histone deacetylation is associated with a “closed” chromatin structure and the repression of gene activity [28]. Histone acetyltransferases and histone deacetylases are responsible for the reversible acetylation of histones (Table 1). At the outset, attention centered on identifying enzymes responsible for the introduction (“writers”) and removal (“erasers”) of these modifications [28]. Among these enzymatic agents, there are those that add chemical groups to histone tails or core domains, such as HATs, kinases, methyltransferases, and ubiquitinases. In contrast, there are enzymes that eliminate these modifications, including HDMs, phosphatases, deubiquitinases, and HDACs. Histone acetylation and deacetylation are critical regulators of plant stress tolerance. In *Arabidopsis thaliana*, a model plant, the HD2-type histone deacetylase HD2C has been found to negatively regulate drought tolerance. HD2C represses the expression of drought-responsive genes by deacetylating histones in their promoter regions, leading to reduced gene expression and impaired drought response in the plant [29]. Conversely, in Arabidopsis, certain genes positively regulate salt stress tolerance by enhancing the expression of salt-responsive genes. As an example, HD2C and HDA6 (histone deacetylase) work in tandem to govern the reaction to salt stress by controlling the expression of ABA-responsive genes including *ABSCISIC ACID INSENSITIVE1* and *ABSCISIC ACID INSENSITIVE2*. The heightened expression of HDA705, a counterpart of Arabidopsis’ HDA6 or HDA7, led to diminished ABA levels and reduced salt stress tolerance in Arabidopsis seedlings [30]. Histone acetylation patterns also impact heat stress responses in wheat (*Triticum aestivum*). *TaHAG1* is a gene that encodes a histone acetyltransferase that is orthologous to Arabidopsis *AtHAG1/GCN5,* and rice *OsHAG702* promotes heat stress tolerance in wheat [31]. Moreover, histone acetylation can also modulate plant defense responses against pathogens. In Arabidopsis, the histone acetyltransferase *HAG1* (HISTONE ACETYLTRANSFERASE OF THE GNAT FAMILY 1) is involved in activating defense genes against bacterial pathogens. HAG1 acetylates histones in the promoters of these genes, promoting their expression and enhancing the plant’s resistance to bacterial infection [32]. Furthermore, epigenetic memory and priming have been observed in maize (*Zea mays*), where exposure to brief drought stress induces changes in histone acetylation patterns, leading to improved drought tolerance upon subsequent stress [33,34].

### 3.2. Methylation and Demethylation

The equilibrium of a specific covalent histone modification’s steady-state level is governed by a delicate interplay between enzymes that facilitate its addition and those that facilitate its removal. Protein arginine methyl transferases (PRMTs) and histone lysine methyl transferases (KMTs) mark lysine and arginine with methyl groups, respectively. Histone lysine methylation occurs primarily at Lys4, Lys9, Lys27, and Lys36 of H3 in Arabidopsis [69,70,71]. Overall, histone methylation at H3K9 and H3K27 is connected with gene silencing, whereas methylation at H3K4 and H3K36 is tied to gene activation. Based on the number of methyl groups added to histone molecules, methylation is classified as mono-, di-, or trimethylation, and gene expression varies depending on the level of modification [26]. For instance, in Arabidopsis, the trimethylation of Lys27 (H3K27me3) leads to gene expression repression, while the trimethylation of Lys4 (H3K4me3) leads to the activation of gene transcription [72]. These methylation marks can be eliminated by histone demethylases (HDMs) with the assistance of various cofactors in plants, including lysine-specific demethylase 1 (LSD1) and the Jumonji C domain-containing protein (JMJ) [73,74,75] (as shown in Table 1). Methylation occurs on lysine and/or arginine amino acids within histones, altering their interaction with reader proteins and consequently influencing chromatin structure, which in turn determines whether transcription is activated or repressed. In Arabidopsis, repressive histone methylation modifications, such as H4R3me2, H3K9me2/3, and H3K27me3, are observed, whereas active histone methylation modifications, such as H4R3me2, H3K4me3, and H3K36me2/3, are evident [76,77]. Unlike acetylation, which damages the electrostatic properties of histone proteins, histone methylation preserves the electron charge of lysine. The histone methylation mark’s mode of action (Tran’s effects) is presumably coordinated through hydrophobicity; however, this assertion is not absolute, and other hypotheses have been put forth. Moreover, a variety of histone H3 lysine residue methylation holds significance in plants, encompassing repressive dimethylation at Lys9 (H3K9me2) and trimethylation at Lys27 (H3K27me3), along with permissive trimethylation at Lys4 (H3K4me3) and Lys36 (H3K36me3) [78]. Additionally, plants exhibit two arginine methylation sites (H3R17 and H4R3) and five lysine methylation sites (H3K4, H3K9, H3K27, H3K36, and H4R20), each potentially holding a distinctive role in the orchestration of transcriptional regulation [76]. For instance, Polycomb Repressive Complex 2 (PRC2) having an HMT unit mediates the histone modification H3K27me3, which was reported to be associated with gene repression in eukaryotes [79,80]. The identification of these PRC2 complexes originally occurred in Drosophila as Hox gene regulators, subsequently revealing homologous PRC2 subunits within plants and animals [81,82].

Histone demethylation involves the removal of methyl groups from specific lysine or arginine residues on histone proteins. This modification can have a profound impact on chromatin structure and, subsequently, on the transcriptional regulation of genes involved in plant abiotic stress tolerance. Histone demethylation can either activate or suppress the transcription of stress-related genes [83]. The specific effect depends on the histone residue being demethylated and the enzyme responsible for the demethylation. For example, the removal of methyl groups from histone H3 lysine 4 (H3K4) is associated with gene activation, while the demethylation of histone H3 lysine 9 (H3K9) or histone H3 lysine 27 (H3K27) is linked to gene repression [84]. The demethylation of histone H3K4 and histone H3 lysine 36 (H3K36) near the promoter regions of stress-responsive genes can lead to their activation [85]. This allows plants to mount a rapid response to abiotic stress conditions by increasing the expression of genes involved in stress tolerance, such as those encoding heat shock proteins, antioxidant enzymes, and osmoprotectants.

### 3.3. Phosphorylation

Phosphorylation, the process of adding a phosphate group (PO_4_^3−^) to a molecule, is orchestrated by specific protein kinases, while phosphatases facilitate phosphate group removal [86]. Within this complex landscape, histones, the proteins around which DNA is wound in chromatin, are subject to dynamic phosphorylation events that primarily target threonine (Thr), serine (Ser), and tyrosine (Tyr) residues [87]. Histone phosphorylation often responds to signals, such as DNA damage, extracellular cues, or cell division progression. In the context of histone modifications, phosphorylation on histone H3 is of particular interest, with prominent sites including Ser 10, Ser 28 (H3S10ph and H3S28ph), Thr 3, and Thr 11 (H3T3ph and H3T11ph) [88]. The importance of these modifications is underscored by observations in Arabidopsis where a mutant deficient in closely related Ser/Thr protein kinases (At3g03940 and At5g18190) displayed heightened sensitivity to osmotic and salt stress, along with dwarfism. Intriguingly, this mutant exhibited a significantly reduced level of phosphorylated histone H3 at Thr 3 (H3T3ph). Genome-wide assessments unveiled an elevation in H3T3ph at Thr 3 within pericentromeric regions of Arabidopsis thaliana under osmotic stress conditions [89,90,91].

This phosphorylation of histone H3 plays a crucial role in various cellular processes, including chromosome segregation, chromatin condensation, and transcriptional regulation [92]. Additionally, histone H2AX phosphorylation at Ser 129, known as γH2AX, is a pivotal player in the DNA damage response and repair. Rapid phosphorylation of H2AX occurs at sites of double-strand DNA breaks, catalyzed by PI3K kinases. This modification represents one of the earliest and most discernible post-translational signals triggered by DNA damage [93,94,95]. The Arabidopsis genome houses an extensive array of over 1000 protein kinases, including calcium-dependent protein kinases (CPKs), mitogen-activated protein kinases (MAPKs), receptor-like kinases (RLKs), and sucrose nonfermenting-related kinases (SnRKs). Alongside these, it hosts approximately 150 protein phosphatases, encompassing type 1 (PP1) and type 2A phosphatases, the protein tyrosine phosphatase family, and the metal-dependent protein phosphatase family [96]. Specific MAPKs, such as MPK3, MPK4, and MPK6, have been identified as key players in the phosphorylation of HSFA4A at Ser-309. This intricate regulatory mechanism serves to modulate the activity of the heat-activated factor HSFA4A. Elevated temperatures and increased salinity both trigger HSFA4A activation, and their combined action influences the accessibility of HSFA4A-binding sites within the promoters of target genes, like *ZAT12*, *HSP17.6A*, and *WRKY30*. This finely tuned orchestration governs the plant’s response to abiotic stresses [97]. Histone phosphorylation, influenced by the activity of MAPKs and the action of transcription factors, like HSFA4A, can further modulate gene expression. It can make the chromatin structure more permissive or restrictive for the transcription of the genes involved in heat stress response. Overall, the connection among MAPKs, HSFA4A, and histone phosphorylation lies in the signaling pathway activated by heat stress in plants. MAPKs are involved in the early signaling events of heat stress response, phosphorylating HSFA4A, which, in turn, activates the transcription of stress-responsive genes. Histone phosphorylation can then act as an additional layer of regulation, ensuring that the right genes are expressed in response to heat stress, contributing to the plant’s adaption and survival under adverse conditions.

Furthermore, rising temperatures induce the nuclear translocation of the BR-regulated transcription factor, brassinazole-resistant 1 (BZR1). In the nucleus, BZR1 binds to the promoter of PIF4 (phytochrome-interacting factor), leading to cell elongation [98,99]. Notably, histone H2A phosphorylation at Ser 95 in Arabidopsis, catalyzed by MuTP9-like kinases, such as MLK4 and MLK3, has been shown to promote flowering time and enhance the deposition of H2A.Z [92].

In summary, histone phosphorylation is a dynamic and finely regulated process involving a delicate balance between kinases and phosphatases. These kinases engage diverse targets to orchestrate distinct temperature-signaling pathways, thereby governing the plant’s responses to a range of temperatures from elevated to exceedingly high. This intricate regulatory network underscores the pivotal role of histone phosphorylation in plant stress tolerance.

### 3.4. Ubiquitination

The enzymatic process involving the transfer of one or more ubiquitin monomers to the protein substrate is termed ubiquitination (or ubiquitylation) [100]. Monoubiquitination brings about alterations in the subcellular localization, biochemical properties, or molecular functions of target proteins. In contrast, polyubiquitination serves as a signal for proteasome-mediated degradation [101]. This modification occurs specifically on lysine residues 119 of H2A and 120 of H2B. The ubiquitination process primarily encompasses three stages: ubiquitin protein activation, ubiquitin conjugation, and ubiquitin ligation. These steps necessitate the addition of ubiquitin to the target protein and are executed by their respective ubiquitin-activating enzymes (E1s), ubiquitin-conjugating enzymes (E2s), and ubiquitin ligases (E3s) [102]. Among the ubiquitination enzymes, the most abundant are E3 ubiquitin ligases. In rice and arabidopsis, a drought stress tolerance cascade involving 3 ubiquitin ligase OsPUB67 and its target protein OsDIS1 and OsRZP34 has been well explored. OsPUB67 positively regulates drought tolerance by promoting improved scavenging of ROS and closure of stomata under drought, whereas OsDIS1 and OsRZP34 are negative regulators that open the stomata under drought stress. OsPUB67 ubiquitinates the targets OsRZFP34 and OsDIS1 for proteolysis-mediated degradation, leading to an increased level of stomatal closure [103,104,105].

In a study conducted by Tripathi et al. [106], researchers delved into the physiological role of OsNAPL6, a putative rice NAP superfamily histone chaperone responsive to stress. This nuclear-localized histone chaperone possesses the ability to form nucleosome-like structures. Through a combination of overexpression and knockdown strategies, they unveiled a positive connection between OsNAPL6 expression levels and the plant’s ability to adapt to diverse abiotic stresses. Their investigation, involving comparative transcriptome profiling and promoter recruitment analyses, highlighted OsNAPL6′s role in stress response by influencing the expression of various genes associated with diverse functions. Many ubiquitin ligases were discovered in stress-related mutants, accounting for regulatory roles in abiotic stress tolerance, particularly drought tolerance, in Arabidopsis and crop species [107]. For example, a small regulatory protein (E3 ubiquitin ligase RING FINGER 1) imparts drought tolerance in durum wheat [1]. Furthermore, H2A (H2Aub) and H2B (H2Bub) monoubiquitination affect transcription in eukaryotes both actively and repressively. In Arabidopsis, H2AK121 monoubiquitination occurs independently of H3K27me3, but it does not cooperate with PRC2, which is required to maintain H3K27me3 [108,109].

In addition to DNA methylation, histone H3 heterochromatic methylation is required for H2B deubiquitination. H2B monoubiquitination, in more detail, activates transcription via the presence of H3K4me3 [110]. The experimental study in Arabidopsis observed a direct link between H2B monoubiquitination and plant immunity because they found that pathogen infection increases the H2B monoubiquitination at R-gene *SNC1* [111]. Likewise, in tomatoes, the presence of the histone H2B monoubiquitination enzymes HUB1, HUB2, SlHUB1, and SlHUB2 has been identified as a contributor to resistance against *B. cinerea*. Their role is likely centered on maintaining a balance between the signaling pathways governed by SA and JA/ethylene. The expression of the gene governing the SA (salicylic acid)-mediated signaling pathway was significantly upregulated, while the expression of genes in the JA (Jasmonic acid)/ethylene pathway were critically downregulated. This interaction establishes a crucial connection between plant immunity and the process of ubiquitination [112]. According to research on wheat’s histone modification, TaHUB2 (the second histone H2B monoubiquitination enzyme) interacts with TaH2B in vernalization pathways and may be necessary for wheat heading [113,114]. TaHUB2 serves as a ubiquitin RING-type E3 ligase. In Arabidopsis, the HUB1 (histone monoubiquitination 1, gene encoding E3 ligase) mediated H2Bub1 (histone H2B monoubiquitination) has been proven as a mechanism regulating auxin biosynthesis [115]. A recent investigation revealed that GhUbox8, an E3 ligase of the U-box-type, collaboratively modulates histone monoubiquitination of H2A and H2B in conjunction with GhUBC2L, an E2 enzyme. This concerted action governs the expression of genes associated with cell cycle progression and organ development [116]. This discovery reinforces the significance of histone monoubiquitination in orchestrating the regulation of organ size within the context of cotton.

### 3.5. Sumoylation

The Small Ubiquitin-like Modifier (SUMO) protein family engages in a process of attaching to and detaching from various proteins within the cell, thereby modulating the functionality of these proteins. In the research conducted by Shiio et al. [117], it was demonstrated that SUMO can modify H4, leading to the recruitment of HDAC and HP1 proteins. This recruitment subsequently results in the suppression of transcriptional activity through competitive interactions with other active marks, such as methylation, acetylation, and ubiquitination. In Arabidopsis, there are instances of SUMOylated chromatin modifiers and components, such as HDA19, H2B, and GCN5, and the deubiquitinating enzyme UBP26, which functions to remove ubiquitin attached to H2B. As an illustration, exposure to heat stress (37 °C for 30 min) induces a reduction in H2B SUMOylation while simultaneously increasing SUMOylation in GCN5 HAT [118]. This phenomenon plays a pivotal role in modulating DNA methylation patterns during heat stress within Arabidopsis, as the SUMOylation of histone acetylases/deacetylases facilitates the conversion of euchromatic regions into heterochromatic ones [119]. In the Arabidopsis context, SUMOylation associated with chromatin serves as a pivotal switch, regulating the transcriptional balance between plant development and the response to heat stress. These SUMO-mediated changes in chromatin signals lead to the upregulation of heat-responsive genes and the downregulation of growth-related genes. Notably, the inactivation of the SUMO ligase gene *SIZ1* resulted in reduced SUMO signals on chromatin and a corresponding attenuation of rapid transcriptional responses to heat stress [120,121].

Through a comprehensive approach encompassing proteomic and interactome analyses, a total of 350 SUMO targets and SUMO-interacting proteins were unveiled in Arabidopsis. This exploration extended to those entities that exhibited accumulation subsequent to subjecting plants to conditions of heat and oxidative stress, employing three distinct research methodologies [118,122]. The majority of SUMO substrates control nuclear activities, such as DNA methylation, DNA repair, RNA processing, chromatin remodeling, and gene transcription [123,124]. Genes, such as *COP1* and *SIZ1,* serve as typical examples of proteins that dynamically regulate high-temperature-induced growth responses via SUMOylation or ubiquitination. Alternatively, SUMO can be subjected to controlled proteolysis via the generation of poly-SUMO chains mediated by SUMO ligases like PIAL1/2. This process is paralleled by the regulation of SUMO through the activity of SUMO ligases, such as PIAL1/2, which orchestrate the assembly of poly-SUMO chains. This polymeric configuration serves as a docking site for STUbLs, facilitating the conjugation of polyubiquitin chains to both SUMO and the target protein. Consequently, this orchestrated interaction primes the 26S proteasome for targeted degradation. This intricate interplay elevates the scope of transcriptional control to a greater level [125].

## 4. Tools to Study Histone Modifications

In terms of research methodology, scientists studying plants must continue to adopt cutting-edge genomics tools that permit genome-scale readouts in addition to single-cell-type studies of gene expression and chromatin profiling. Technological advances have provided suitable techniques to investigate the chromatin profiles and gene expressions in particular plant cell types.

### 4.1. Chromatin Immunoprecipitation (ChIP) and ChIP-qPCR

**Figure 3:**
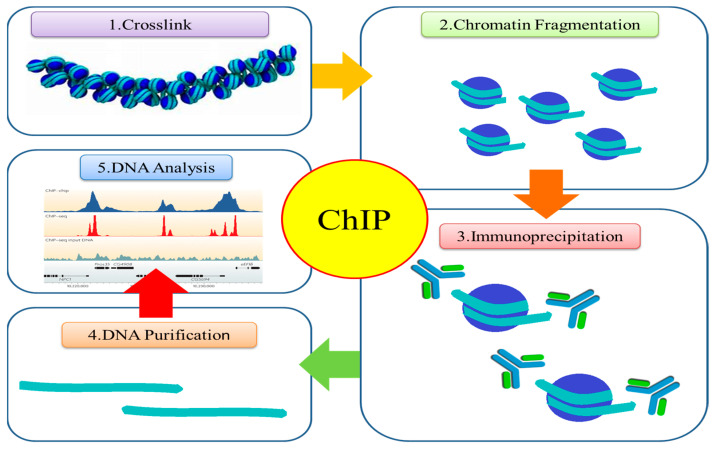
Overview of a ChIP experiment. Formaldehyde is used to cross-link proteins and their associated chromatin in living cells. In the next step, cross-linked DNA–protein complexes (chromatin–protein) are physically or enzymatically digested to separate them into 500 bp DNA fragments. In immunoprecipitation, an appropriate protein-specific antibody is subsequently used to immunoprecipitate the DNA–protein complexes. The related DNA fragments are eluted after the cross-links are reversed, and then the cross-linked complexes are immunoprecipitated. The resultant DNA is subjected to analysis through using endpoint or quantitative polymerase chain reaction (qPCR), microarray technology (ChIP-chip), or state-of-the-art next-generation sequencing (ChIP-seq).

The significance of chromatin regulation in plant stress responses has been underscored through the discovery of distinct sites of histone modification and the identification of histone modifiers that control critical stress-responsive genes using both genetic and biochemical approaches [87]. The intimate connection between histone modifications and the ChIP technique lies in the fact that ChIP (Chromatin Immunoprecipitation) serves as the foremost experimental tool for investigating and delineating histone modifications at specific genomic loci. Using the ChIP technique, scientists can examine how histone proteins that have particular post-translational modifications bind to DNA, revealing important details about the epigenetic control of gene expression. ChIP stands as a potent and adaptable technique, centered on the selective immunoprecipitation of a protein of interest from chromatin extracts. This method serves the purpose of elucidating the DNA sequences linked with the protein, and it has emerged as the preferred approach for scrutinizing protein–DNA interactions within cellular contexts. Its utility extends to the mapping of DNA target sites for transcription factors and other proteins linked to chromosomes [126,127,128], as well as for pinpointing the genomic positioning of post-translationally modified histones and histone variants (as illustrated in Figure 3).

So far, many ChIP experiments in plants have been conducted. ChIP analyses, for example, have identified 28 histone modification sites in *Arabidopsis* [129]. According to the results of a ChIP assay conducted in *Arabidopsis* for a drought–stress response, the activation of four distinct drought stress-responsive genes is accompanied by changes in histone modifications on the histone 3 N-terminal tails, such as *At2g20880*, *RD20*, *RD29A*, and *RD29B*. Another finding from the ChIP assays indicates that the factor ERF1 activates particular sets of genes in response to different abiotic stresses through stress-specific binding to DRE/CRT or GCC. Furthermore, a study reported that the expression of *At*ERF53 (Ethylene Response factor 53) in wild-type plants of Arabidopsis results in responsive behavior of those plants to ABA and heat stress, which implicates the positive role of *At*ERF53 in the transactivation of downstream stress-related genes [130]. Based on a ChIP-PCR investigation, it has been revealed that the promoter region of the *MIR168a* gene undergoes direct binding by four distinct autonomously replicating sequence binding factors (ABF1, ABF2, ABF3, and ABF4) in response to ABA or drought treatment [10]. Furthermore, the integration of ChIP with quantitative PCR (ChIP-qPCR) or sequencing (ChIP-seq) has emerged as a highly effective approach for pinpointing histone modifications at specific loci or across the entire genome. This methodology is also instrumental in identifying sites where DNA-binding proteins (DBPs) are active [131,132]. In summary, ChIP allows researchers to assess changes in histone modifications under abiotic stress by selectively isolating and analyzing DNA regions associated with specific histone modifications. This technique helps elucidate the epigenetic regulation of genes involved in stress responses and provides insights into the molecular mechanisms underlying how organisms adapt to challenging environmental conditions [133,134].

Following the ChIP assay and sample purification, qPCR enables real-time quantification of DNA concentrations from multiple samples by examining fluorescent signal intensities that are proportional to the amount of amplicon. With the help of primers, polymerases, oligonucleotides, and detection fluorophores, like TaqMan (fluorescent donor: quencher hybridization) probes, or SYBR Green intercalating dye (no specific probe is needed), DNA samples are subjected to quantitative PCR. In optimum reactions, DNA polymerase doubles the amount of amplified DNA through cycles of amplification. Each cycle uses products from the previous cycle as templates for the subsequent cycle. In order to be certain of a successful analysis, it is important to make sure that the primers amplify the targeted sequence with greater than 95% efficiency and do not form dimers, which could weaken the specific signal from SYBR Green-based qPCR [135,136]. This approach finds utility across various domains in the life sciences, encompassing cellular differentiation, the suppression of tumor suppressor genes, and the influence of histone modifications on gene expression. A Chip-qPCR protocol has been established to discern and evaluate histone modifications within youthful Arabidopsis inflorescence tissue [137].

### 4.2. Bisulphite-Treated Chromatin-Immunoprecipitated DNA (BisChIP-seq)

**Figure 4:**
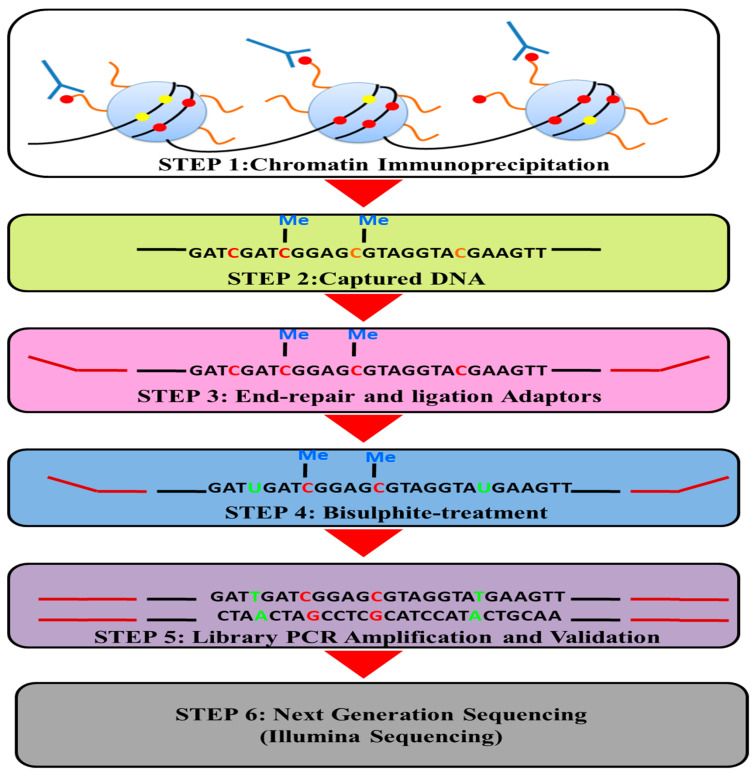
Bisulphite sequencing of chromatin-immunoprecipitated DNA (BisChIP-seq) to obtain information on the methylation status of histone-modified DNA.

Researchers are deciphering the intricate patterns of DNA methylation and histone modifications within chromatin, both in the context of cellular differentiation during development and in the context of diseases, through the utilization of extensive whole-genome sequencing endeavors [138]. Moreover, the implementation of bisulphite sequencing on chromatin-immunoprecipitated DNA (BISChIP-seq) provides a direct means to explore whether DNA marked by histones or associated with transcription factors is methylated. Bisulphite sequencing involves treating DNA with bisulphite to convert cytosine, while preserving 5-methylcytosine (5mC), into uracil. Subsequent PCR amplification results in the conversion of uracil into thymine. ChIP is used to enrich a target chromatin mark in order to achieve this. As a result of successful ChIP, the DNA undergoes end repair, 30 ends are adenylated, and methylated adaptors are ligated before bisulphite conversion is completed. It is necessary to convert the ChIP DNA to achieve the best DNA yield and the highest bisulphite conversion rates (>98%). In BisChIP-seq, library generation is the final step, and by using PCR amplification of adaptor-ligated bisulphite-converted DNA over 10–14 cycles of PCR, a library is generated, which is then subjected to next-generation sequencing [139] (Figure 4).

This technology enables accurate qualitative and semiquantitative assessments of DNA methylation. Consequently, it becomes a valuable tool for discerning variations in DNA methylation levels within specific genes under both normal and stress-inducing conditions. Through direct analysis of bisulfite methylation in wild tobacco plants, an insightful mapping emerged, indicating complete demethylation within promoter regions and targeted demethylation specifically occurring at CG sites within coding regions. These observed phenomena had previously been linked to oxidative stress, as demonstrated by the comparable demethylation pattern induced by ROS triggered by paraquat exposure. As an illustration, the aluminum treatment of wild tobacco plant leaves prompted the induction of NtGPDL transcripts within a mere 6 h timeframe. The bisulphite sequencing of such plants exposed to abiotic stress revealed a cause–effect relationship between NtGPDL methylation and expression. Also, in another study, Bisulphite sequencing has been performed on rice genotypes [10].

### 4.3. Cleavage under Targets and Tagmentation (CUT & Tag)

Cleavage Under Targets and Tagmentation (CUT & Tag) is an innovative approach to epigenome profiling. In this method, a specific antibody binds the target chromatin protein directly in its native context. Subsequently, the antibody serves as a bridge to the pA/G-Tn5 transposase fusion protein. Activation of the pA/G-Tn5 transposase in the presence of Mg^2+^ initiates a ‘cut-and-paste’ process on the target chromatin. As a result, the target DNA becomes fragmented and is tagged with adaptor sequences, a step referred to as tagmentation [140]. CUT & Tag is designed to target specific genomic regions or proteins, including histones with specific modifications. For instance, researchers can use antibodies against histone modifications of interest (e.g., H3K4me3 for active promoters) to selectively profile these modifications in response to abiotic stress [141]. It can also generates high-resolution maps of histone modifications, allowing researchers to pinpoint precisely where these modifications occur in the genome. This is essential for understanding the regulatory elements and genes associated with stress responses.

While CUT & Tag techniques have demonstrated success in profiling histone modifications, investigating specific transcription factor (TF)–DNA interactions using CUT & Tag remains technically demanding primarily because TFs are typically lower in number [142,143,144,145].

In summary, CUT & Tag is a versatile epigenomic profiling technique that can be applied to study histone modifications in plants under abiotic stress conditions. Its ability to provide high-resolution, cell-specific data makes it a valuable tool for understanding the epigenetic regulation of stress-responsive genes and pathways in plants.

### 4.4. Mass Spectrometry

Histone post-translational modifications (PTMs) are commonly examined through using mass spectrometry (MS), especially when seeking to uncover unexplored PTMs that may represent novel targets. The “bottom-up” MS approach, valuable for discerning proteins within intricate mixtures and performing peptide mapping of purified proteins, involves enzymatic protein digestion into smaller peptides and subsequent liquid chromatography (LC) separation and MS detection ensue. Due to the abundant basic residues in histones, the use of trypsin-based bottom-up MS that primarily focuses on lysine and arginine, generates peptides that are often very low for comprehensive characterization of histone PTMs. To comprehensively capture the intricate interplay of combinatorial PTMs spanning the polypeptide sequence, enhanced protocols have been devised. These involve chemical derivatization or alternative proteases, such as GluC instead of trypsin, resulting in the generation of longer peptides [146,147]. These elongated peptides are better-suited for Liquid Chromatography–Mass Spectrometry (LC–MS) characterization. Employing the top-down MS approach proves highly effective for profiling histone modifications and distinguishing closely related forms of histone proteins. Prior investigations utilizing top-down MS have successfully unveiled novel variants, PTMs, and proteolytic sites [109,148,149]. The utility of top-down MS is progressively expanding across diverse biological applications [150].

## 5. Role of PTM-Based Histone Modifications in Abiotic Stress Tolerance

Abiotic stresses, such as drought, extreme temperatures, salinity, and heavy metal exposure, induce various changes in plant cells, including alterations in the epigenetic landscape through histone modifications. When plants encounter these stresses, they activate a complex network of signaling pathways and stress-responsive genes to adapt and survive. Histone modifications play a pivotal role in regulating gene expression under these adverse conditions [151,152]. For example, the acetylation of histone proteins provides a more open chromatin structure, facilitating access to stress-responsive genes for transcription factors and RNA polymerase [153,154]. Conversely, methylation and deacetylation events can lead to gene silencing, providing a mechanism for the downregulation of nonessential genes during stress. These modifications are orchestrated by histone-modifying enzymes and epigenetic regulators, and can mediate both short-term responses to acute stress and long-term adaptations to chronic stress conditions [155,156,157]. Overall, histone modifications represent a crucial layer of epigenetic regulation in plants, enabling them to fine-tune gene expression in response to various abiotic stresses, ultimately promoting survival and adaptation in challenging environments.

**Figure 5:**
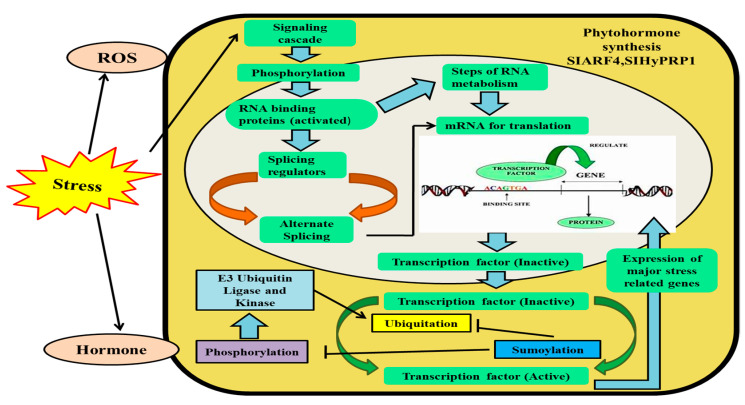
Post-translational changes are controlled by sophisticated molecular mechanisms in plant cells against abiotic stress. Specific receptors sense external stresses and transduce signals to the internal components, which in turn activate plant defense mechanism through post-translational modifications.

Extreme temperatures, droughts, high salinity, and ultraviolet radiation, all of which are brought on by unpredictable climate changes, have a significant negative impact on plant productivity. In response to environmental stresses, histone modifications play an important role in regulating gene expressions as depicted in (Figure 5).

### 5.1. Role in Heat Stress

Sessile plants are vulnerable to heat stress, a pivotal abiotic stressor profoundly restricting plant growth and diminishing yields [158]. To enable plants to endure and counteract heat stress, a specific subset of heat-responsive genes necessitates either activation or suppression. This regulatory process can be orchestrated through histone or chromatin modifications [159,160]. Illustratively, GCN5 emerges as a key player in bolstering thermotolerance by enhancing the acetylation of H3K9/K14 within the promoter regions of genes, such as *ULTRAVIOLET HYPERSENSITIVE6* and *HEAT SHOCK TRANSCRIPTION FACTOR A3*. Notably, plants harboring gcn5 mutations exhibited marked deficiencies in thermotolerance upon exposure to heat stress [64]. While nonenzymatic acetylation reactions remain a plausible phenomenon, the prevalence of such reactions in plant nucleosomes remains uncharted territory. Recent studies have unveiled an array of acetylation sites on nonhistone proteins, with several of these sites proving pivotal for abiotic stress response [161]. Notably, these findings underscore the significance of enzymatic acetylation catalyzed by HAT activity in enabling Arabidopsis to withstand abiotic stress. However, it is worth highlighting that, particularly in the context of histone H3 acetylation, GCN5/HAG1 primarily targets H3, with only marginal effects on H4 or H2A/B acetylation in vitro. Furthermore, the interplay between H3K4 demethylation and histone H3K9 acetylation triggers the upregulation of Hsf (Heat Stress Transcription Factor), facilitating elevated ROS levels that ultimately aid in the acclimatization of maize seedlings to heat stress [162].

The outcomes reveal that heat stress-responsive genes, including *ATX1* and *SDG25*, exhibit expression in Arabidopsis both during heat stress and in the subsequent stress recovery phase. Furthermore, the overexpression of RING finger E3 ligases, such as OsHTAS, OsHIRP1, or OsHCI1, has been demonstrated to bestow heat tolerance upon rice plants [120,163,164]. As detailed in a study conducted by Mishra et al. [66], the augmentation of the rice SUMO E3 ligase OsSIZ1 through overexpression notably escalated the photosynthesis rate in plants experiencing heat stress. Notably, when rice plants encountered moderate heat stress at 48 and 72 h postfertilization, a decline was observed in the levels of DNA and H3K9 methylation associated with OsFIE1 (Fertilization-Independent Endosperm 1). This observation hints at the role of epigenetic regulation in influencing thermosensitivity during endosperm development [165]. These findings hold the potential to unveil novel perspectives on enhancing thermotolerance in rice. Arabidopsis ASF1 (ANTISILENCING FUNCTION 1) histone chaperones, encompassing ASF1A and ASF1B, have been associated with both innate and acquired thermotolerance, facilitated by nucleosome eviction and the promotion of H3K56ac.

The transcriptional induction of *HSFA2* and *HSA32* genes underscores the pivotal role of histone modification as the principal mechanism orchestrating the plant’s response to heat stress [166]. As highlighted by Qiu et al. [167], plants respond to prolonged slightly elevated temperatures primarily through thermal morphogenesis, which encompasses phenomena, like petiole elongation and leaf hypophysis. Notably, the overexpression of SUMO E3 ligases (such as OsSIZ1, GmSIZ1, AtSIZ1, and SlSIZ1) has demonstrated a capacity akin to ubiquitination in enhancing plant resilience to heat stress [65,66,168]. Newer investigations have revealed that SUMO1/2 activation contributes to temperature acclimation through the HSFA1 family, recognized as the principal orchestrator of heat stress responses. Notably, mutant lines lacking SUMO1/2 exhibit thermosensitivity at 28 °C [169]. In a recent discovery by Zhang et al. [168], an additional E3 ligase, the XB3 ortholog in Arabidopsis thaliana (XBAT31), has been identified. This E3 ligase is implicated in regulating the stability of the ELF3 protein, consequently impacting hypocotyl growth under elevated temperatures. Another insightful study on rice unveiled the significance of OsbZIP74 in conferring heat stress tolerance. This study highlighted that OsNTL3, a NAC transcription factor that governs downstream gene expression in response to heat and ER stress, experiences heightened gene expression levels due to the influence of OsbZIP74 [170].

### 5.2. Role in Cold Stress

Plant development and growth can be significantly impacted by cold stress. Extremely low temperatures (chilling and freezing) can cause chlorosis, wilting, uncontrolled apoptosis, and damage to the photosynthesis process in plants [171]. However, plants have developed special systems to cope with cold stress; among them, epigenetic regulations play an important role [172]. As detailed in the study by Zeng et al. [25], plants react to cold stress by enhancing the accessibility of active gene chromatin and imprinting it with a bivalent pattern. Building upon their observations, the researchers put forward a hypothesis that the bivalent marks of H3K4me3-H3K27me3 in cold-stored tubers create a unique chromatin milieu with heightened accessibility. This environment could potentially aid in facilitating the regulation of genes involved in the response to cold stress.

One of the distinctive mechanisms is referred to as the cold signaling mediated by the Cold-Responsive Element-Binding Factors (CBFs; also known as Dehydration-Responsive Element-Binding Protein (DREB): CBF1/DREB1B, CBF2/DREB1C, and CBF3/DREB1A) transcription factors. These CBF transcription factors play a pivotal role in inducing the expression of specific genes associated with freezing resistance. They achieve this by binding to the conserved Cold-Responsive Element/Dehydration-Responsive Element (CRT/DRE) present in the promoter region of Cold-Regulated genes (COR), such as *COR15A*, *COR47*, and *COR78*. This process contributes significantly to the enhancement of freezing tolerance. In Arabidopsis, exposure to extremely low temperatures triggers an increase in the expressions of CBF2 and CBF3 (c-repeat binding factors) through the overexpression of RDM4 (RNA-directed DNA Methylation-4). This enhanced expression of CBF2 and CBF3 contributes to a reduction in membrane injury by interacting with COR (cold-related) genes [173,174]. In a study by Min et al. [175], it was observed that transgenic rice plants exhibiting ectopic overexpression of CaPUB1, a U-box E3 ubiquitin ligase from hot pepper, displayed heightened resistance to cold stress compared to wild-type rice plants. Another gene, *HOS15* (*HIGH EXPRESSION OF OSMOTICALLY RESPONSIVE GENE15*), plays a distinct role in mediating HD2C degradation upon exposure to cold stress. This action alters the chromatin structure from inhibitory to active, serving as a positive regulator of cold stress [28]. Consequently, this regulatory mechanism enhances CBF supplementation, leading to the development of cold hardiness and the expression of COR genes. Additional investigations revealed that cold stress exposure in potato tubers triggers bivalent histone modifications (H3K4me3 and H3K27me3), leading to enhanced chromatin accessibility [176]. For cold tolerance, Hwarari et al. [177] demonstrated that the CBF gene protein engages with the CRT/DRE, a conserved regulatory element within the promoter of the COR gene.

### 5.3. Role in Salt Stress

Elevated soil salt concentrations exert detrimental effects on plants, resulting in diminished plant growth and development. To contend with salt stress, plants employ epigenetic mechanisms, such as chromatin remodeling and histone modifications for genetic-level regulation [178,179]. Notably, an increase in the H3K9 acetylation levels has been correlated with the upregulation of cell wall-related genes in maize roots, essential for an effective response to high salinity. Prominent examples of these genes encompass the promoter and coding regions of ZmXET1 and ZmEXPB2. Despite the amplified mRNA expression triggered by salt stress, the potential involvement of two HAT genes (*ZmHATB* and *ZmGCN5*) in orchestrating this upregulation has been suggested. Recent investigations revealed the role of GCN5 in imparting heat and salinity stress tolerance. In the context of salt stress response, the involvement of GCN5 was initially discerned in maize (*Zea mays*) roots. The heightened acetylation of H3K9, manifesting in both gene promoter and coding regions, was associated with the upregulation of cell wall-related genes, such as *ZmEXPB2* and *ZmXET1*, enabling maize roots to effectively counteract high-salinity conditions. This acetylation-driven response is believed to be orchestrated by two histone acetyltransferase (HAT) genes, *ZmHATB* and *ZmGCN5*, whose mRNA expression escalates in the presence of salt stress [180]. Likewise, in Arabidopsis, GCN5 mRNA expression becomes activated under salt stress, and gcn5 mutants show higher susceptibility to salt stress due to compromised cell wall integrity, corroborating earlier findings. A critical GCN5 target crucial for both cell wall biosynthesis and salt stress resilience is CTL1, which encodes a chitinase-like (CTL) protein. GCN5′s acetylation of H3K9/K14 serves as a stimulant for CTL1 expression, thereby bolstering cell wall synthesis and enhancing salt stress resistance [176].

Another important finding was the isolation of the AP2/EREBP TF family member HhBREB2 from the *Halimodendron halodendron* and subsequent assignment of this gene to the DREB subfamily’s A-5 cluster due to its similarity to the AP2/ERF domain. Increased salt tolerance was produced in Arabidopsis by overexpressing the *HhBREB2* gene [181] that has shown promise in mitigating salinity-induced stress in cassava plants. By utilizing suberoylanilide hydroxamic acid (SAHA), a commercially available HDAC inhibitor, researchers have endeavored to enhance cassava’s resilience to high-salinity conditions. Notably, exposure to SAHA led to a robust upregulation of an enzyme involved in jasmonic acid (JA) biosynthesis. This, in turn, contributed to a reduction in Na^+^ levels and an increase in K^+^/Na^+^ ratios. Microarray-based transcriptome analysis unveiled a pronounced induction of ABA and JA phytohormone biosynthesis in response to heightened salinity stress. Immunoblotting analyses provided evidence of SAHA treatment triggering extensive hyperacetylation of histones H3 and H4 in cassava roots, confirming the inhibitory role of SAHA as an HDAC inhibitor. Transcriptome analysis further revealed that under normal conditions, SAHA enhanced the expression of 421 root genes, a number that increased to 745 genes at 2 h and 268 genes at 24 h during SAHA and NaCl treatment. The primary outcome of SAHA treatment was the hyperacetylation of histones H3 and H4 in cassava roots, potentially instigating transcriptional modifications that enhance the plant’s tolerance to high salinity stress.

For the involvement of specific HDACs, it has been observed that HDA9 and HDA19 negatively impact salt stress tolerance, while HDA6, HD2C, and HD2D exert a positive influence on salinity resilience. In a comparable context, rice INDETERMINATE SPIKELET1 (IDS1) has been identified as an HDAC recruiter in salinity stress [38]. Furthermore, in Arabidopsis, ubiquitination at histones H2A and H2B has been linked to both transcriptional activation and repression [182,183]. In accordance with Dong et al. [184], the epigenetic landscape of the promoter region of *MsMYB4*, a gene encoding a salt-induced MYB transcription factor in alfalfa, undergoes changes in response to salt stress. The activation of *MsMYB4*, critical for alfalfa’s salt stress response, was correlated with elevated levels of histone H3K4 trimethylation and H3K9 acetylation within specific segments of the promoter sequence. In the case of rice, *HDA710*/*OsHDAC2* has been observed to modulate salt stress-responsive genes by influencing the acetylation status of H4 within its promoter regions, as evidenced by the notable rise in HDA710 transcript levels under salt stress conditions [185]. In wheat, a wheat counterpart of *AtHAG1*/*GCN5* known as *TaHAG1* has been identified to directly target genes associated with ROS synthesis. This targeting was found to be crucial for wheat’s adaptation to salt stress, as the enrichment of *TaHAG1* during salt stress led to increased H3 acetylation and subsequent transcriptional upregulation of these genes [186].

### 5.4. Role in Drought Stress

Drought exerts significant adverse impacts on crop yield, emerging as a paramount agricultural concern. With the projections of intensified and more frequent global climate change, the incidence and severity of drought are anticipated to escalate [17,187]. Under drought stress, mRNA expression of genes, like *OsHAC703*, *OsHAF701*, *OsHAG703*, and *OsHAM701,* in rice was increased [45]. Similarly, investigations revealed that the *O. sativa* arsenic-induced RING E3 ligase 1 (OsAIR1) and the *O. sativa* chloroplast-targeting RING E3 ligase 1 (OsCTR1) acted as positive regulators of the drought stress response [188]. Notably, small noncoding RNAs (sncRNAs) displayed a positive correlation with hypermethylated regions across three distinct rice cultivars with varying degrees of drought tolerance, highlighting an ongoing interplay among small RNA abundance, gene expression, and DNA methylation during stress conditions [189]. To illustrate, in Chinese cabbage, drought stress led to a decreased expression of *BraHAC5*, while it notably induced strong upregulation in the expression of *Bra-HAC7*, *BraHAG2*, and *BraHAC5*. Interestingly, after the drought stress treatments, *Bra-HAG2* exhibited a contrasting expression pattern between days 2 and 4. In the context of salinity stress, it has been demonstrated that 13 BraHATs were activated at different time points (5 h, 1 day, and 2 days) following the stress treatments, except for *BraHAG4* and *BraHAG6* [44]. For instance, rice Indeterminate Spikelet1 (IDS1) and Arabidopsis MYB96 have been identified as HDAC recruiters in the context of salinity and drought stress responses, respectively [38,39]. In poplar (*Populus trichocarpa*), Abscisic Acid (ABA)-Responsive Element-Binding Protein1 (AREB1) functions as a HAT recruiter during the drought stress response [36]. Conversely, in rice, OsbZIP46CA1 (OsbZIP46) serves as both a recruiter for H2B ubiquitination and deubiquitination in the context of the drought stress response [61].

Under drought stress conditions, the tomato Heat Shock Transcription Factor A1a (SlHsfA1a) engages with the promoters of *SlATG18f* and *SlATG10*, triggering activation in response to the drought stress stimulus [62]. Subsequently, Bao et al. [190] unveiled the Arabidopsis gene Constitutively Stressed 1 (*AtCOST1*), which governs autophagy and manages plant drought tolerance. In Arabidopsis, the protein Salt- And Drought-Induced Ring Finger1 (SDIR1) positively modulates the ABA-mediated drought stress response [67,191]. Rice plants also possess OsSDIR1, an ortholog of SDIR1, involved in orchestrating the drought stress response [192]. Cui et al. [193] unveiled and characterized *Oryza sativa* Drought-Induced RING Protein 1 (OsDIRP1), a potential RING-type E3 Ub ligase present in rice. Both drought and high-salinity conditions triggered the induction of OsDIRP1. Furthermore, under drought stress, there was an increase in H3K4me3 and H3K9ac at the promoter regions of stress-responsive genes, such as *RD29A*, *RD29B*, *RD20*, and *RAP2.4* in Arabidopsis [194]. In a separate study, the examination of histone deacetylase 9 (HDA9) of Arabidopsis thaliana with RPD3-type characteristics was conducted [46].

### 5.5. Role in UV and Radiation Stress

Although plants utilize sunlight during photosynthesis, excessive light, particularly its UV (ultra-violet) component, can stress cells and cause serious harm to DNA, proteins, and other constituents. Plants have been demonstrated to develop “memory” based on epigenetic changes against UV stress [195]. The UV-B stress also induces ROS production causing metabolic impairment, decreased resistance to pathogens, and alterations in cellular processes [196,197,198]. In the early stages, distinct variations in the observed histone modifications were detected at specific loci in Arabidopsis seedlings under varying light conditions. The investigation revealed that shifts between dark and light environments could bring about alterations in the H3K9ac status, which is additionally influenced by the quality and quantity of light. Interestingly, key regulators in UV signaling and photomorphogenesis, namely HY5, DET1, and COP1, appear to be involved in modulating H3K9ac levels. Notably, the H3K9ac status of specific genes showed a strong correlation with their transcription in mutant forms of these three proteins. Additionally, exposure to UV-B increased the H3K9/K14 acetylation on the ELIP1 (Early Light-Inducible Protein 1) promoter in wheat and Arabidopsis. The UV-C-exposed Arabidopsis plants were observed to inherit the stress tolerance to untreated genotypes via methylation of the genome and homologous frequency [199].

Lang-Mladek et al. [200] documented heritable variations in Arabidopsis in response to UV-B stress, affecting the epigenetic regulation of a reporter gene. These changes were associated with modifications in histone H3 acetylation and alterations in chromatin conformation. Chromatin modifications were observed in maize seedlings subjected to UV-C and gamma irradiation treatments, and a clear connection has been established among resistance to UV-C exposure, germination rate, and the occurrence of chromosomal aberrations [201]. Researchers examined the histone modifications linked to DNA damage repair genes in Arabidopsis seedlings exposed to gamma rays [202]. Following gamma irradiation, there was an elevation in the transcription of DNA damage repair genes. Investigations have demonstrated that the heightened expression of these genes is associated with histone H3K4me3 at the gene body or transcription start sites. This conclusion was drawn from diverse chromatin modification analyses performed at specific loci using the conventional ChIP procedure [203]. Various investigations have unveiled the impact of DNA methylation analysis at different levels within the loci of At1g31280 (AtAgo2), At4g19130 (AtRPA1E), At5g20850 (AtRad51), and At5g24280 (AtGMI1). This analysis was carried out through using MethylC-Seq (bisulphite conversion followed by sequencing) using genomic DNA extracted from 4-week-old soil-grown plants. The outcomes of these studies led researchers to a notable finding: the expression of DNA damage repair genes increased significantly after exposure to gamma irradiation [184,204,205]. Additionally, specific regions of the promoter sequences exhibited histone modifications, namely H3K4me3 and H3K9ac. H3K4me3 was observed at gamma ray-inducible loci during DNA damage repair, while H3K9ac was identified exclusively at the AtAgo2 locus. Remarkably, the research unveiled that histone modifications triggered by gamma radiation are site-specific. Furthermore, the modifications H3K4me3 and H3K9ac seem to play a more crucial role in the activation of gamma radiation-induced genes at these specific loci than in the alterations in DNA methylation [206,207].

## 6. Limitations and Path Ahead

Climate change presents arduous tasks to plants for survival and adaptation, to which plants respond by frequent adjustments to adverse environments. Consequently, recent research has emphasized understanding the role of PTMs in the molecular regulation of stress-related genes. PTMs in histones offer plants greater flexibility in activating their enzyme-signaling pathways, expediting resistance compared to post-transcriptional processes. This review provides detailed clues on the significance of histone PTMs and chromatin remodeling in governing stress responses and genetic regulation in plants.

Researchers have achieved great success in resolving the mysterious role of histone PTMs in abiotic stress tolerance, but shaping the future of stress-tolerant plant development requires addressing certain limitations in diagnosing histone PTMs. Firstly, the challenge of proteome complexity is a significant hurdle. The intricate landscape of PTMs results in a diverse range of proteoforms contributing to functional diversity within a single gene product. Proteoforms (protein variants) refer to various isoforms of histone proteins that arise due to specific combinations and locations of PTMs on histone proteins. These protein variants play a critical role in the epigenetic regulation of gene expression and the dynamic control of chromatic structure within the cell. This complexity poses challenges for accurate identification, demanding advanced techniques capable of capturing the dynamic nature of histone PTMs. While progress has been made in mass spectrometry and high-throughput methods, cataloguing the entire spectrum of proteoforms (protein variants) remains challenging. Consequently, achieving a comprehensive understanding of how PTMs orchestrate gene regulation and plant responses to abiotic stresses remains a formidable task, necessitating ongoing innovation in analytical approaches. Moreover, deciphering the functional consequences of specific protein variants is another limitation. Elucidating the significance of individual variants requires sophisticated experimental approaches, including targeted mutagenesis, functional assays, and in-depth structural analyses. The complex interplay between different PTMs within a single protein further complicates the interpretation of their combined effects on protein function.

Exploring this field has the potential to enhance our capacity to intricately intertwine regulatory pathways with precise epigenetic adjustments, encompassing enhancer and promoter specificity, RNA polymerase processivity, and pre-mRNA splice site selection. The utilization of emerging, precise genome-editing techniques is poised to offer ample opportunities to explore the application of targeted histone modifications in addressing various abiotic stresses [208]. This proactive approach has the potential to significantly advance our strategies for managing multiple stressors in plant systems.

Furthermore, understanding these histone PTMs will greatly enhance our ability to explore the level of detail and precision at which individual cells are studied or analyzed within a biological sample, often referred to as single-cell resolution [209]. The future of studying PTMs in plant abiotic stress tolerance at single-cell resolution lies in harnessing advanced single-cell omics technologies, including scRNA-seq, single-cell proteomics, and spatial transcriptomics, to dissect the intricacies of PTM dynamics within individual plant cells. By doing so, this approach will unveil cell-specific responses to abiotic stressors, identify key regulatory nodes, and enable the development of predictive PTM signatures for abiotic stress tolerance. Furthermore, the understanding of histone PTMs will be significantly boosted by research advances in plant stress memory [210].

Future perspectives of PTMs in climate resiliency involve the continual refinement of analytical strategies, the integration of multiomics approaches, and the development of bioinformatics tools to decipher the functional significance of protein diversity. Employing network analyses and systems biology approaches can reveal the convoluted relationships between proteoforms and other cellular components, uncovering regulatory networks and signaling pathways for stress resilience.

## Figures and Tables

**Table 1:** Histone modifications occurring under different abiotic stresses in plants. Special attention is given to the proteins acting as substrates for modifications, encompassing both histone and nonhistone proteins.

Modification Type	Regulator Name	Crop	Stress Type	References
Acetylation Acetyltransferase	*GCN5*, *AtHAC1*	Arabidopsis and Poplar	Heat, salinity, and drought (Chimeric dCas9 HAT)	[35,36,37]
Acetylation Acetyltransferase	HAT, AREB	Poplar	Drought	[36]
Deacetylation (Deactylase)	HDAC, IDS1	Rice	Salinity	[38]
Deactylation (Deactylase)	HDAC, MYB96	Arabidopsis	Drought	[39]
Deactylation Deacetylase	HDA9, HDA15, HDA705, BdHD1, HD2C	Arabidopsis, Rice, and Brachypodium	Drought, salinity cold, and heat	[28,39,40,41,42]
H3K9 acetylation	HAT, *GCN5*, *ZmEXPANSIN-B2*	Maize	Salinity	[43]
H3 hyperacetylation	*HAT* genes, *OsHAT* genes	Rice	Drought	[44,45]
Deactylation (Deactylase)	HDA9, CYP707A1, CYP707A2	Arabidopsis	Drought	[46]
Deactylation (Deactylase)	BdHD1, WRKY24	Purple False Brome or Stiff Brome	Drought	[47]
Acetylation	*AtHAC1*	Arabidopsis	Heat	[48]
Acetylation	MYST, ELP3, *GCN5*	Barley	Drought	[49]
Acetylation	*OsHAC703*, *OsHAG703*, *OsHAF701*, *OsHAM70*	Rice	Drought	[50]
Deacetylation (Deactylase)	84KHDA903	Tobacco	Drought	[51]
Deacetylation (Deactylase)	HD2C, HSFA3, HSFC1, HSP10	Arabidopsis	Heat	[42]
Acetylation	*GCN5*, PtrNAC006,	Black Cottonwood Tree	Drought	[52]
Recruiter	MYB96, IDS1, AREB1	Arabidopsis, Rice, and Poplar	Drought and salinity	[38,39,52]
Methylation Methyltransferase	ATX1, ATX4/5	Arabidopsis	Drought	[53,54]
Demethylation Demethylase	JMJ17	Arabidopsis	Drought	[55]
Trimethylation	HMT	Arabidopsis	Gamma irradiation	[56]
Ubiquitination Ubiquitinase	HUB1/2, AtHUB2, OsHUB2	Arabidopsis, Cotton, and Rice	Salinity and drought	[57,58,59,60,61]
Phosphorylation Kinase	MLK1/2	Arabidopsis	Drought and salinity	[62,63,64]
Ubiquitinase and deubiquitinase	H2B	Rice	Drought	[61]
Sumoylation	SUMO E3 ligase (AtSIZ1, OsSIZ1)	Arabidopsis and Rice	Heat	[65,66,67]
Ubiquitination	*SNAC1* gene	Wheat	Salt and drought	[68]

## Data Availability

No new data were created or analyzed in this study. Data sharing is not applicable to this article.

## Abbreviations of Genes

(c-repeat binding factors)	CBF2 and CBF3
84KHDA903	Tobacco Histone Deacetylase
AP2/EREBP TF	APETALA2/Ethylene-responsive Element-Binding Protein Transcription Factor
AP2/ERF	APETALA2/Ethylene-responsive Factor
AREB	ABA-Responsive Element-Binding Protein
ASF1	Antisilencing Function 1
At1g31280 (AtAgo2)	Argonaute 2
At2g20880	ERF53 Drought-Induced Transcription Factor
At3g03940 and At5g18190	Ser/Thr protein kinases
At4g19130 (AtRPA1E)	Replication Factor-A protein 1-like protein
At5g20850 (AtRad51)	Homolog of Yeast RAD (Radiation repair gene) 51
At5g24280 (AtGMI1)	Gamma Irradiation and Mitomycin C-Induced 1
AtCOST1	Arabidopsis gene Constitutively Stressed 1
AtHAG1	*Arabidopsis Thaliana* Histone Acetyltransferase
ATX1, ATX4/5	Arabidopsis homolog of Trithorax1,4 and 5 (histone methyltransferase)
BdHD1	*Brachypodium distachyon* Histone Deacetylase
BraHAC5	Histone Acetyltransferase of the CBP Family 5
BraHAG2	Histone Acetyltransferase of the GNAT Family 2
BZR1	Brassinazole-resistant 1
CBFs	Cold-Responsive Element-Binding Factors
CMT3	Chromomethylase 3
Cold-regulated genes (COR), such as COR15A, COR47, and COR78	
COP1	Constitutive Photomorphogenic 1
CPKs	Calcium-Dependent Protein Kinases
CRT/DRE	Cold-Responsive Element/Dehydration Responsive Element
CTL	Chitinase-like
CYP707A1, CYP707A2	Cytochrome P450 family 707-ABA 8′-Hydroxylase
DET1	De-etiolated 1
DOGL4	Delay of Germination 1-LIKE 4
DRE/CRT	Dehydration-responsive element/C-repeat
DREB	Dehydration-Responsive Element-Binding Protein
DRM	Domain rearrangement methylase
DSB	Double-Strand Break
ELP3	Elongator Acetyltransferase Complex Subunit 3
ERF113	Ethylene-Responsive Factor113
GCN5	General Control nondepressible 5
GNAT-MYST	Gcn5-related N-acetyltransferase
HAG1	Histone Acetyltransferase of the Gnat Family 1
HATs	Histone Acetyltransferases
HDACs	Histone Deacetylases
HDMs	Histone Demethylases
HhBREB2	Halimodendron halodendron
HMTs	Histone Methyltransferases
HOS15	High Expression of Osmotically Responsive Gene15
HSFA3/A4A	Heat Shock Transcription Factor A3/A4A
HSFC1	Heat Shock Transcription Factor C1
HSP10/HSP17.6A	Heat Shock Protein 10/17.6A
HUB1/2, AtHUB2,OsHUB2, SlHUB1, SlHUB2	Histone Monoubiquitination1/2
HY5	Elongated Hypocotyl 5,
IDS	Iduronate 2-Sulfatase
IDS1	Indeterminate Spikelet1
JMJ17	Jumonji Domain-Containing Protein 17
LBD-16	Lateral Organ Boundaries Domain
MAPKs	Mitogen-Activated Protein Kinases
MET1	Methyltransferase 1
MLK1/2	MUT9p-Like Kinase1/2
MPK3/4/6	Mitogen-Activated Protein Kinase 3/4/6
MYB96	myb Domain Protein 96
MYST	MOZ, Ybf2/Sas3, Sas2, and Tip60
MYST	MYST-Type Domain-Containing Lysine Acetyltransferase
OsAIR1	*Oryza sativa* arsenic-induced RING E3 ligase 1
OsbZIP46CA1	Basic leucine zipper Transcription Factor
OsbZIP74	Leucine Zipper Transcription Factor
OsCTR	*Oryza sativa* chloroplast targeting RING E3 ligase 1
OsDIRP1	*Oryza sativa* Drought-Induced RING Protein 1
OsDIS1	*Oryza Sativa* E3 ubiquitin–protein ligase 1
OsHAC703, OsHAF701, OsHAG703, and OsHAM701	
OsHAG702	*Oryza sativa* Histone Acetyltransferase
OsHCl1	Ring Finger E3 Ligase
OsHIRP1	Heat-Induced Ring Finger protein
OsHTAS	Heat Tolerance at Seedling Stage
OsNAPL6	*Oryza Sativa* histone chaperone of NAP superfamily
OsNTL3	Membrane-Associated NAC Transcription Factor
OsPUB67	*Oryza Sativa* U-box E3 ubiquitin ligase
OsRZP34	*Oryza Sativa* Ring Zinc Finger Protein 34
PIAL1/2	Protein inhibitor of activated STAT-like 1
PP1	Protein Phosphatases1
PRC2	Polycomb-Repressive Complex 2
PtrNAC006	*Populus truchocarpa* NAC Domain-Containing Protein 6
RAP2.6	Related to AP2 6
RD20/29A/29B	Responsive to Desiccation 20/29A/29B
RDM4	(RNA-directed DNA Methylation-4).
RLKs	Receptor-Like Kinases
RTS1	Repressor of Transcriptional Silencing 1
SDIR1	Salt- and Drought-Induced Ring Finger1
SIARF4	*Solanum lycopersicum* Auxin-Response Factor 4
SIHyPRP1	*Solanum lycopersicum* Hybrid Proline Rich Protein 1
SIZ1/OsSIZ1/GmSIZ1/AtSIZ1/SlSIZ1	SAP and MIZ 1 Domain containing Ligase 1
SlHsfA1a	Heat Shock Transcription Factor A1a
SNAC1	Stress-responsive NAC1 gene
SNC1	Suppressor of NPR-1, Constitutive1
SnRKs	Sucrose Nonfermenting-Related Kinases
WIND1	Wound-Induced Dedifferentiation 1
WRKY24/30	*Arabidopsis thaliana* WRKY DNA-Binding Protein 24/30
XBAT31	XB3 Ortholog 1in *Arabidopsis thaliana*
ZAT12	Zinc Finger of *Arabidopsis thaliana* 12
ZmEXPB2	Zea maize expansin-B2
ZmXET1	Zm Xyloglucan Endotransglucosylase Hydrolase 1
